# Classical 11β-Hydroxylase Deficiency Caused by a Novel Homozygous Mutation: A Case Study and Literature Review

**DOI:** 10.7759/cureus.21537

**Published:** 2022-01-23

**Authors:** Mohammad N Alsanea, Abdulmoein Al-Agha, Mohamed Abdelmaksoud Shazly

**Affiliations:** 1 Internal Medicine, King Abdulaziz University Faculty of Medicine, Jeddah, SAU; 2 Pediatrics, King Abdulaziz University Faculty of Medicine, Jeddah, SAU

**Keywords:** atypical genitalia, hypertension, hyperplasia, adrenal, congenital

## Abstract

Congenital adrenal hyperplasia (CAH) is an uncommon condition and 11β-hydroxylase deficiency (11βOHD) accounts for 0.2-8% of cases. In this study, we report a three-year-old girl with a known diagnosis of classical CAH on maintenance treatment with hydrocortisone who presented with abnormal genitalia and persistent hypertension. Genetic testing confirmed the diagnosis of autosomal recessive CAH due to 11βOHD as a result of a novel homozygous pathogenic mutation, c.53dup p.(Gln19Alafs*21), in the *CYP11B1* gene. Physicians should consider the possibility of classical 11βOHD in CAH patients presenting with persistent hypertension, even if other laboratory biomarkers are equivocal.

## Introduction

Congenital adrenal hyperplasia (CAH) is a genetic endocrine metabolic disease characterized by autosomal recessive defects in one of the adrenal enzymes responsible for glucocorticoid biosynthesis [1,2]. It was first discovered by De Crecchio in 1865 [3]. The spectrum of clinical presentations of CAH depends on the defective enzyme and the severity of the defect. Clinical manifestations stem from both the failure to synthesize hormones distal to the enzymatic block and from the accumulation of cortisol precursors proximal to the block, often with a shift to other biologically active steroids [2]. CAH leads to diminished production of cortisol and decreased or increased production of mineralocorticoids and/or androgens depending on the site of the block. Patients with CAH show a wide spectrum of clinical presentations depending on the underlying enzyme deficiency, including deficiency of 21-hydroxylase, 11β-hydroxylase, 3β-hydroxysteroid dehydrogenase, and 17α-hydroxylase [4]. The majority of CAH cases (90-99%) occur due to 21-hydroxylase deficiency, while 11β-hydroxylase deficiency (11βOHD) accounts for only 0.2-8% of cases [5,6]. In this study, we report a case of congenital adrenal hyperplasia due to 11βOHD in a three-year-old girl who presented with abnormal genitalia and hypertension.

## Case presentation

**Figure 1:**
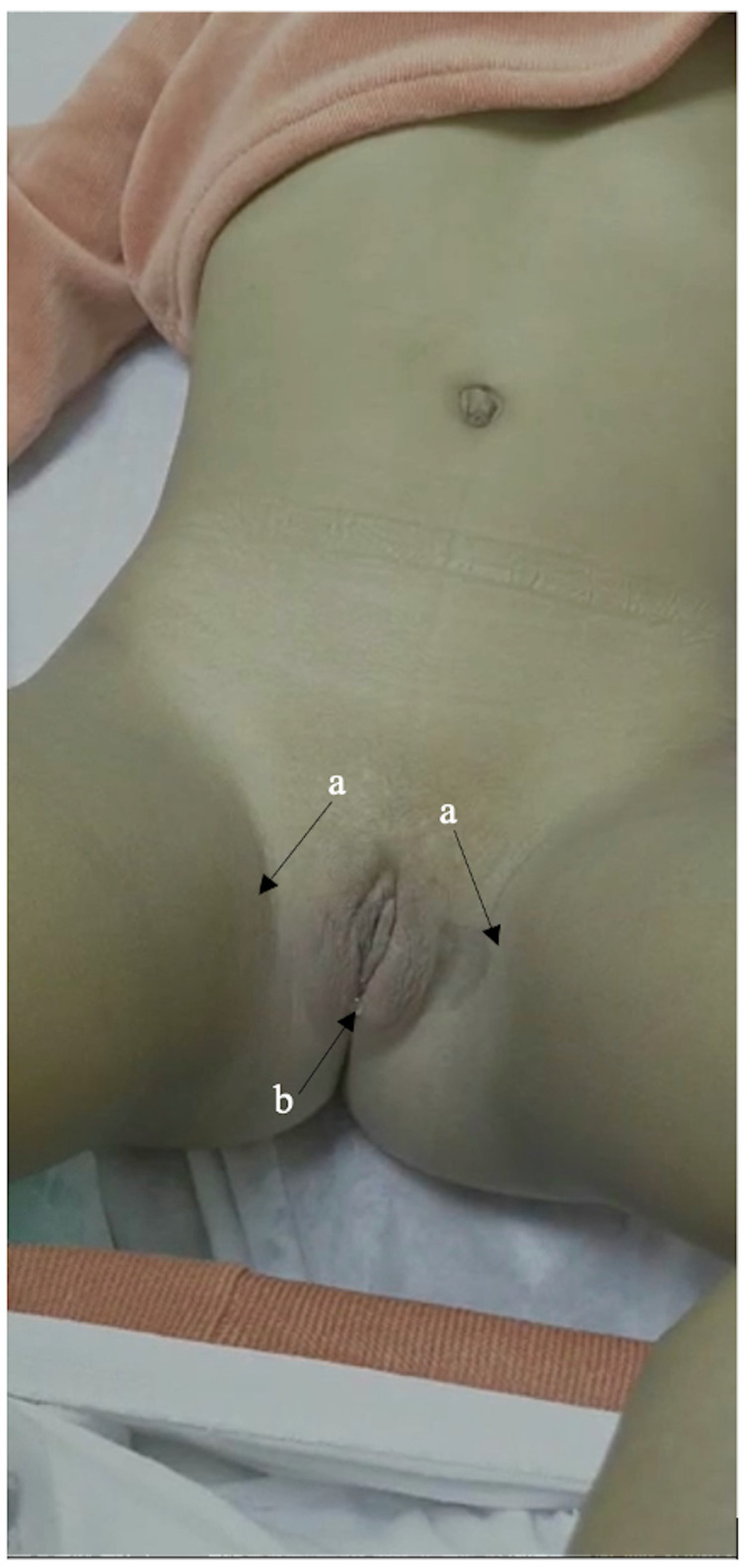
Genitalia of the patient (46,XX) showing (a) hyperpigmentation, (b) bifid labioscrotal folds, a single urogenital sinus with an enlarged phallus, and no palpable gonads.

A three-year-old girl from Yemen with a known diagnosis of classical congenital adrenal hyperplasia on maintenance treatment with hydrocortisone 2.5 mg thrice daily. She was brought to the Pediatric Endocrine Clinic because of repeated episodes of hypertension for six months duration. Her review of systems and past medical history were insignificant and unremarkable. Her antenatal and natal histories were normal. She was born at full term via spontaneous vaginal delivery with a birth weight of 3100 g. Upon delivery, abnormal genitalia was observed. Her parents were first-degree cousins and there was no family history of similar conditions. On examination, her height and weight were 103 cm and 16.80 kg, respectively, which were both above the 75th percentile for her age and sex. Her systolic and diastolic blood pressure average measurements were 164 and 91 mmHg, respectively, heart rate was 110 beats/min, temperature was 36.8 °C, and respiratory rate was 22 breaths/min. She had no dysmorphic features. Systemic examination normal except for hyperpigmented elbows and knees; however, genital examination revealed hyperpigmentation, bifid labioscrotal folds, a single urogenital sinus with an enlarged phallus, and no palpable gonads (Figure 1). She had no signs of hyperandrogenism. The laboratory results are shown in Table 1.

**Table 1:** Laboratory analysis of the patient while she was on her maintenance replacement therapy. BUN: blood urea nitrogen

Parameter	Test Result	Reference Range
Hemoglobin	11.9 g/dL	10.2–15.2 g/dL
Leukocytes	7.87 K/uL	5–17 K/uL
Platelets	406 K/uL	150–450 K/uL
Blood Glucose	4.6 mmol/L	3.9–6.7 mmol/L
Adrenocorticotropic hormone	4.1 Pmol/L	1.6–13.9 Pmol/L
Morning Cortisol (8 AM)	288.35 nmol/L	140–690 nmol/L
Renin	1.58	0.6 to 4.3 ng/mL/h
Aldosterone	2.67	2-9 ng/dl
Testosterone	1.25 nmol/L	0.42–2.06 nmol/L
Dehydroepiandrosterone sulfate	4.70 umol/L	1.65–11.60 umol/L
Androstenedione	13.2 ng/dL	5–51 ng/dL
17-Hydroxy-progesterone	11.7 ng/dL	4–115 ng/dL
Estradiol	43.32 Pmol/L	26–125 Pmol/L
Follicle-stimulating hormone	1.31 IU/L	1.5 to 33.4 IU/L
Luteinizing hormone	0.10 mIU/L	0.5–76.3 mIU/L
Sodium	139 mmol/L	136–145 mmol/L
Potassium	3.6 mmol/L	3.5–5.1 mmol/L
Chloride	107 mmol/L	98–107 mmol/L
Urea (BUN)	2.2 mmol/L	2.5–6.4 mmol/L
Creatinine	30 umol/L	53–115 umol/L
Urine analysis	Negative	Negative
Urine culture	Negative	Negative

Imaging modalities were used to rule out other associated abnormalities. Renal ultrasonography showed normal kidneys and internal female reproductive organs with a small prepubertal uterus and hydrocolpos with possible inferior vaginal stenosis at the level of the urogenital sinus. A voiding cystourethrogram (VCUG) showed no evidence of vesicoureteral reflux. The results of the other systemic investigations were normal. Chromosomal analysis revealed a normal karyotype of 46,XX and genetic testing, which was previously ordered upon her presentation in the clinic, confirmed the diagnosis of autosomal recessive CAH due to 11βOHD as a result of a homozygous pathogenic mutation, c.53dup p.(Gln19Alafs*21), in the *CYP11B1* gene. She was commenced on an angiotensin-converting-enzyme inhibitor (Captopril) 6.5 mg thrice daily with good response, bringing her blood pressure average measurements back to the normal range after four weeks.

## Discussion

The worldwide incidence of CAH due to 11βOHD is approximately 1:100,000 live births in the general non-consanguineous population [7]. The incidence of 11βOHD varies geographically, with most cases occurring in the Middle East and North Africa [8]. Moreover, 11βOHD constitutes up to 25% of CAH cases in Saudi Arabia and up to 8% in most other populations [9,10]. Of the 11βOHD cases, 58% are a consequence of consanguineous marriages [8]. Classical CAH patients present with salt-wasting or simple virilizing form at birth or in the neonatal period [4]. Notably, there are no definite criteria to differentiate between all types of CAH; this factor increases the diagnostic challenges associated with such disorders. CAH should be considered as a spectrum of phenotypes, ranging from asymptomatic to severe [11]. The enzymatic deficiency of 11β-hydroxylase reduces the conversion of 11-deoxycortisol (S) and 11-deoxycorticosterone (DOC) to cortisol and corticosterone, leading to their accumulation and shunting into androgens [1]. Classic 11βOHD presents with features of hyperandrogenism, such as virilization of the external genitalia in female newborns, peripheral precocious puberty, and advanced bone age due to premature epiphyseal closure [1,5,7]. Moreover, elevation in the levels of mineralocorticoid-like precursors, such as DOC, leads to the development of mild to moderate hyporeninemic hypertension in two-thirds of the cases at the time of diagnosis, sodium retention, hypokalemia, and acidosis [12-15]; other features include hirsutism, acne, and hyperpigmentation [16]. Our patient had persistent hypertension despite being on hydrocortisone for her CAH. Moreover, she did not present with any classic metabolic abnormalities associated with CAH. The novel genetic mutation we discovered has not been reported previously, and the diagnosis of classic 11βOHD CAH was confirmed. Other known mutations causing classic 11βOHD CAH are summarized in Table 2. The presence of refractory hypertension seems to be a distinctive finding in CAH due to classical 11βOHD, regardless of the presence or absence of other classical biochemical features of CAH [13,17-19]. Recognition and treatment of the underlying cause of hypertension are important because it can lead to retinopathy, left ventricular hypertrophy, intracranial aneurysms, and cerebrovascular disease [10,20-22]. Physicians should be aware that persistent hypertension may be the only presenting symptom of the 11βOHD type of CAH. 

**Table 2:** Summary of genetic mutations causing classic 11βOHD CAH. 11βOHD: 11β-hydroxylase deficiency; CAH: congenital adrenal hyperplasia

Classic 11βOHD		
Mutation	Clinical presentation or notes	Reference
c.954G > A;p.Thr318Thr	Hypertension, severe virilization	Kandemir et al. [23]
p.Arg141*	Hypertension, severe virilization	Kandemir et al. [23] Solyom et al. [24] Zhang M et al. [25]
p.Leu299Pro	Severe virilization	Kandemir et al. [23]
p.His125Thrfs*8	Macrogenitalia, no hypertension	Polat S et al. [26]
p.Leu463_Leu464dup	Testicular adrenal rest tumor	Polat S et al. [26]
p.G379V, p.Q356X	Found in Tunisian population	Kharrat M et al. [27]
IVS7+1G>A	Uniparental disomy	Matsubara K et al. [28]
R448H Non-classic 11βOHD	Moroccan Jews	White PC et al. [29]
p.(Arg143Trp)	Premature pubarche, accelerated growth	Menabò S et al. [30]
p.(Arg332Gln)	Acne, accelerated growth	Menabò S et al. [30]
p.(Ser150Leu)	Premature pubarche, absent virilization	Polat S et al. [26]
p.(Gly446Ser)	Premature pubarche	Kandemir et al. [23]
p.F79I; p.R138C	Premature pubarche, high–normal blood pressure	Reisch N et al. [19]
p.R143W	Hirsutism, primary amenorrhea	Reisch N et al. [19]
p.P159L	Premature pubarche, accelerated growth	Parajes S et al. [18]
p.M88I; p.R383Q	Peripheral precocious puberty	Parajes S et al. [18]
p.R366C	Hirsutism	Parajes S et al. [18]
p.T401A	Accelerated growth	Parajes S et al. [18]
p.P42S	Acne, precocious adrenarche	Joehrer K et al. [31]
p.N133H	Precocious adrenarche	Joehrer K et al. [31]
p.T319M	Acne, precocious adrenarche	Joehrer K et al. [31]

## Conclusions

The present study reports the case of a three-year-old patient with classical 11βOHD presenting with persistent hypertension and carrying a novel homozygous mutation in the *CYP11B1* gene. Persistent hypertension in patients with CAH can be a distinguishing clinical feature for differentiating 11βOHD from other types of CAH. In this case, we highlight the presence of persistent hypertension in the absence of classic metabolic derangements as a clue for the diagnosis of classic 11βOHD. The possibility of classical 11βOHD should always be considered in patients with persistent hypertension, even if other laboratory biomarkers are inconsistent with CAH. Such patients should be carefully identified, diagnosed early, and managed appropriately to avoid the unfavorable consequences of longstanding hypertension.
